# Premature Child Restraint System Transitions and Child Opportunity Index Among Emergency Department and Urgent Care Visits in Metropolitan Chicago

**DOI:** 10.1111/acem.70187

**Published:** 2025-11-05

**Authors:** Arthi S. Kozhumam, Mech Frazier, Michelle L. Macy

**Affiliations:** ^1^ Medical Scientist Training Program Northwestern University Feinberg School of Medicine Chicago Illinois USA; ^2^ Mary Ann & J. Milburn Smith Child Health Outcomes, Research and Evaluation Center, Stanley Manne Children's Research Institute Ann & Robert H. Lurie Children's Hospital of Chicago Chicago Illinois USA; ^3^ Digital Scholarship Northwestern University Libraries Evanston Illinois USA; ^4^ Division of Emergency Medicine, Department of Pediatrics Northwestern University Feinberg School of Medicine Chicago Illinois USA

Motor vehicle collisions (MVC) remain a leading cause of death for children younger than 10 in the United States (US). Suboptimal child passenger safety practices contribute to increased risk of severe injury or fatality for children in an MVC [1]. With more than 1,300,000 children under age 13 seen in the emergency department (ED) for evaluation and care of MVC injuries, ED providers are credible experts in the prevention and treatment of injuries. Therefore, the ED setting can be a place of heightened awareness of injury risk and serve as a contact point in the healthcare system to emphasize primary injury prevention strategies with patients and families, including the use of car seats and seat belts [2]. The ED can also play a role in connecting caregivers to community resources.

Household income, caregiver education, race, and ethnicity are associated with suboptimal child passenger safety practices, which include premature transition to a less protective seat, riding unrestrained, or riding in the front seat before age 13 [3]. However, injury prevention research that focuses on individual‐level markers of socioeconomic status (SES) or race and ethnicity overlooks the potential relationship between community‐level resources and safety behaviors. The Child Opportunity Index (COI) 3.0 [4] is a composite measure of 44 indicators related to children's neighborhood opportunity and resource quality based on US census tract location, including social and economic, education, and health and the environment domains.

To increase efficiency and lower the burden of ED‐based primary injury prevention strategies on social workers, nurses and providers, we aimed to explore how community context, as represented by ZIP code‐level COI (1‐very low, to 5‐very high), relates to premature child passenger restraint system (CRS) transitions in Chicago as a potential variable to identify patients for targeted screening and intervention. We expected lower COI to be associated with higher rates of premature transition given prior literature noting a higher prevalence of premature transition for children of lower SES and Black or Hispanic/Latine racial and ethnic backgrounds.

Data were collected through pre‐recruitment screening for the Tiny Cargo, Big Deal! clinical trial (Lurie Children's IRB #2020‐3274). This analysis focused on surveys completed January 2021–August 2022, the period during which demographic questions were available in the screening survey. Screening of caregivers occurred electronically, during or after their 6‐month to 10‐year‐old child's ED or urgent care visit to four locations in Chicago and its surrounding suburban areas [5]. Children living in Cook County, IL ZIP codes (*n* = 1568) were selected for regression analysis, narrowed to city of Chicago ZIP codes (*n* = 60) for mapping.

Child age, weight, and height were extracted from the electronic health record (EHR). The caregiver's demographics (age, highest level of education, and household income); noted CRS used, or “usual” CRS if the child used multiple types of restraints; and child seating location were included from the surveys. Caregiver‐reported home ZIP code was linked to metro‐normed COI levels (very low, low, moderate, high, very high) which were extracted both overall and in the three COI domains (social and economic, education, and health and the environment). Race and ethnicity are excluded from COI calculations to isolate the structural features of neighborhoods and because race and ethnicity are concentrated at low and high ends of COI distributions [4]. We therefore excluded race, ethnicity, and preferred language from our analyses.

Our primary outcome was premature transition, defined as the caregiver‐reported usual CRS being recommended for a child who is larger or older than the study child. For example, a child using a booster seat when they would still fit in a forward‐facing car seat with an internal harness. Caregiver‐reported CRS use was categorized as appropriate, premature transition, or delayed transition using a novel algorithm based on the American Academy of Pediatrics Child Passenger Safety Policy Statement and size limits for typical CRS on the US market [5].

Descriptive statistics were calculated. Premature transition prevalence was determined for each ZIP code, matched to COI 3.0 for 2021. Child‐level bivariate logistic regression models were used to test for associations between COI (reference: very high; predictors: 1–5 level of COI overall, and each COI domain) and premature transitions (binary outcome). Multivariate models were adjusted for demographic factors of caregiver‐reported age (18–24, 25–34, 35–44, 45+) highest level of education attained (high school or below, associate's or some college, Bachelor's or higher), and household income level (< $50,000, $50,000–$99,000, $100,000). Statistical analyses were conducted in RStudio (2025, Posit Software, MA, USA). ArcGIS Pro version 3.4 (2024, Esri, CA, USA) was used to visualize the prevalence of premature transitions and COI levels across study ZIP codes. Using the Getis‐Ord‐Gi* Hot Spot Analysis (HSA) Tool, hotspot analysis focused on ZIP codes within the city of Chicago due to limited sample density from surrounding suburban areas and adjusted for the number of caregivers invited to screen in each ZIP code. HSA identifies clusters of spatially significant high (hot spot) and low (cold spot) values within a geographic area. The Getis‐Ord‐Gi* statistic created via HSA returns a value (*z*‐score) for each dataset feature (i.e., ZIP code), with hot or cold spots at or above 90% confidence being statistically significant, indicating more intense clustering (see Figure 1).

**FIGURE 1:**
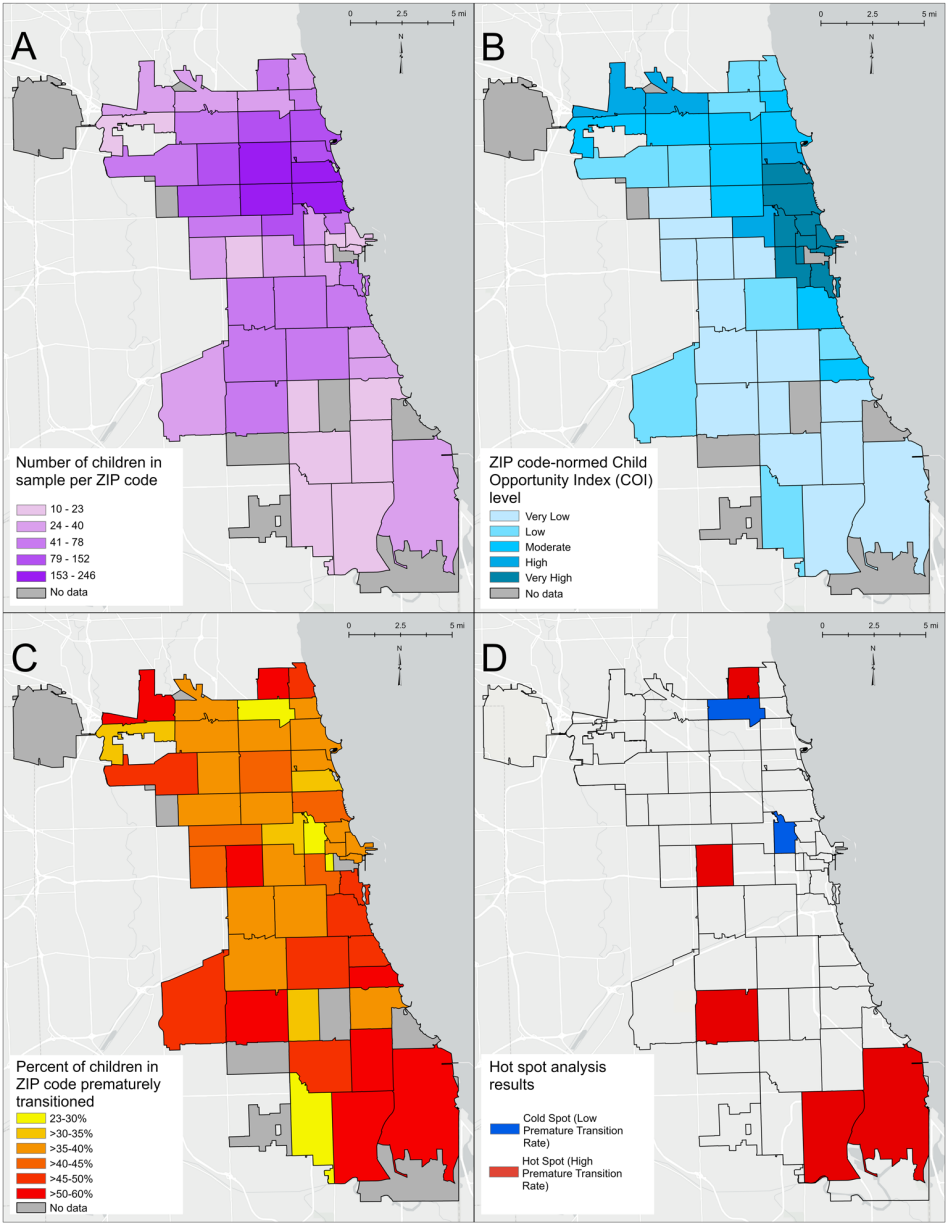
(A) Distribution of premature transitions, (B) Child Opportunity Index Level, (C) sample density, and (D) premature transition density hotspots across ZIP codes in Chicago.

Of 4045 children with screening responses that could be linked to EHR data, 4044 were from 288 ZIP codes in Illinois. Regression analyses included 3996 (98.8%) responses across the 255 ZIPs with available COI level. Most ZIP codes were moderate (*n* = 55, 21.6%) or very high (*n* = 71, 27.8%) COI, followed by high, low (*n* = 47, 18.4% for each) and very low (*n* = 35, 13.7%). Screening survey responses were most common for children in ZIP codes categorized as very high (*n* = 1230, 41.1%) or moderate (*n* = 1012, 33.8%) COI, followed by very low (*n* = 707, 17.7%), low (*n* = 547, 13.7%), and high (*n* = 473, 11.8%) COI (Figure 1A,B).

Nearly one quarter of children overall were prematurely transitioned (*n* = 932, 23.3%), with an inverse relationship between premature transition rates and COI levels: 34.1% (*n* = 241) for very low, 23.9% (*n* = 137) low, 21.0% (*n* = 213) moderate, 20.3% (*n* = 96) high, and 19.2% (*n* = 236) very high COI ZIP codes.

Compared with children from very high COI ZIP codes, those from very low and low COI ZIP codes had higher odds of premature transition in bivariate models (OR 2.18, 95% CI 1.76–2.69 and OR 1.32, 95% CI 1.04–1.67, respectively). Similarly, children from lower COI ZIP codes had higher odds of premature transition across subdomains: social and economic: very low (OR 2.11, 95% CI 1.73–2.59); education: low (OR 1.40, 95% CI 1.12–1.75) and very low (OR 2.26, 95% CI 1.82–2.81); and health and environment: moderate (OR 1.43, 95% CI 1.06–1.97), low (OR 1.42, 95% CI 1.05–1.94), and very low (OR 2.29, 95% CI 1.68–3.15). After adjusting for caregiver demographics, very low overall and education subdomain COI levels remained statistically significant predictors of premature transition (OR 1.31, 95% CI 1.01–1.70 and OR 1.39, 95% CI 1.06–1.81).

Hotspot analyses were limited to 2938 children (73.5%) in 47 Chicago ZIP codes with at least 10 observations and matched to COI level. Higher premature transition ZIP codes generally had lower COI (Figure 1B,C). Five very low and low COI ZIP codes (60,645, 60,624, 60,629, 60,628, and 60,617) were premature transition hotspots and two (60,659, 60,642) were cold spots (Figure 1D).

To our knowledge, this is the first study to examine the association between COI and caregiver‐reported child passenger safety behaviors. We found more than one third of children from very low COI ZIP codes seen in two ED and two urgent care settings in Chicago were prematurely transitioned to a less protective CRS. These findings can shift the field towards the application of community‐level measures of opportunity to guide injury prevention efforts. Prior research has found the ED to be a feasible location for screening and intervention on child passenger safety [6, 7]. Our geospatial approach provides a model for general EDs, pediatric EDs, and urgent care settings to digitally screen for and identify local ZIP codes where children are at greater risk of suboptimal child passenger safety behaviors. Identified locations could be programmed into EHR‐based algorithms to automate flagging of children at greatest risk for premature transition and achieve greater efficiency in primary injury prevention efforts being implemented in a busy ED environment [2]. These locations can also provide targets for the distribution of community resources (e.g., education and CRS) to families from areas of greatest risk for premature transitions [8].

Our main limitation is that premature transitions among children receiving emergency and urgent care in Chicago may not be representative of CRS practices for children in other locations, and children seeking acute care may overrepresent suboptimal practices within a given ZIP code. Further, there is potential for CRS misclassification due to social desirability bias which could underestimate the true premature transition rates. Together, our findings support an opportunity for acute care settings to contribute to screening and precision provision of child passenger safety resources [9].

## Author Contributions

Arthi S. Kozhumam conceptualized and designed the study, carried out the initial analyses, drafted the initial manuscript, and critically reviewed and revised the manuscript. Mech Frazier supervised analyses, and critically reviewed and revised the manuscript. Michelle L. Macy conceptualized and designed the study, designed the data collection instruments, coordinated and supervised data collection, supervised analyses, and critically reviewed and revised the manuscript for important intellectual content. All authors approved the manuscript as submitted and agree to be accountable for all aspects of the work.

## Conflicts of Interest

The authors declare no conflicts of interest.

## Data Availability

The data that support the findings of this study are available from the corresponding author upon reasonable request.
